# Influence of Acetaminophen on Molecular Adsorption and Transport Properties at Colloidal Liposome Surfaces Studied by Second Harmonic Generation Spectroscopy

**DOI:** 10.1021/acs.langmuir.2c00086

**Published:** 2022-03-17

**Authors:** Asela S. Dikkumbura, Alexandra V. Aucoin, Rasidah O. Ali, Aliyah Dalier, Dylan W. Gilbert, Gerald J. Schneider, Louis H. Haber

**Affiliations:** † Department of Chemistry, Louisiana State University, Baton Rouge, Louisiana 70803, United States; ‡ Department of Physics and Astronomy, Louisiana State University, Baton Rouge, Louisiana 70803, United States; § Southeastern Louisiana University, Hammond, Louisiana 70402, United States

## Abstract

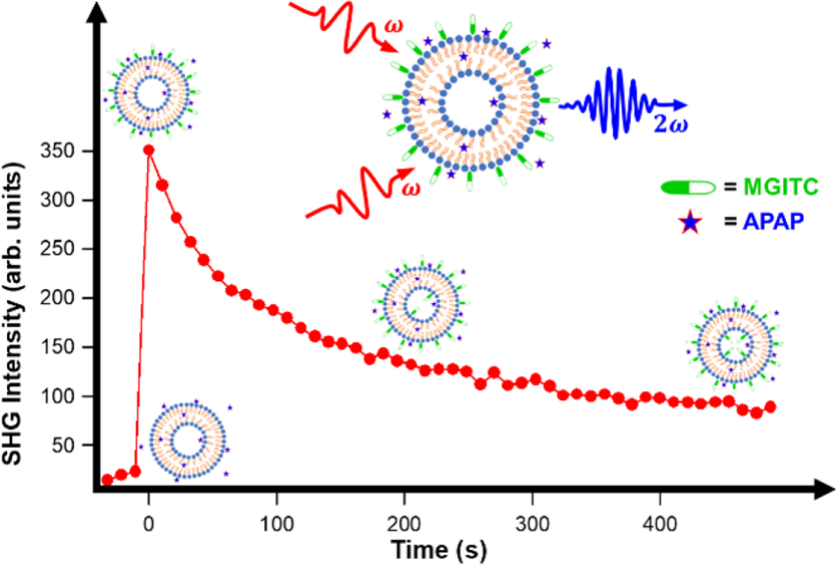

Time-resolved second harmonic generation (SHG) spectroscopy is used to investigate acetaminophen (APAP)-induced changes in the adsorption and transport properties of malachite green isothiocyanate (MGITC) dye to the surface of unilamellar 1,2-dioleoyl-*sn*-glycero-3-phosphocholine (DOPC) liposomes in an aqueous colloidal suspension. The adsorption of MGITC to DOPC liposome nanoparticles in water is driven by electrostatic and dipole–dipole interactions between the positively charged MGITC molecules and the zwitterionic phospholipid membranes. The SHG intensity increases as the added MGITC dye concentration is increased, reaching a maximum as the MGITC adsorbate at the DOPC bilayer interface approaches a saturation value. The experimental adsorption isotherms are fit using the modified Langmuir model to obtain the adsorption free energies, adsorption equilibrium constants, and the adsorbate site densities to the DOPC liposomes both with and without APAP. The addition of APAP is shown to increase MGITC adsorption to the liposome interface, resulting in a larger adsorption equilibrium constant and a higher adsorption site density. The MGITC transport times are also measured, showing that APAP decreases the transport rate across the DOPC liposome bilayer, especially at higher MGITC concentrations. Studying molecular interactions at the colloidal liposome interface using SHG spectroscopy provides a detailed foundation for developing potential liposome-based drug-delivery systems.

## Introduction

Liposomes are closed spherical vesicles consisting of one or more phospholipid bilayers in which drug molecules can be stored.^1,2^ Liposomes are also considered as cell-membrane mimics because they can be composed of the same phospholipids found in the plasma membranes of cells.^3−7^ Because of their excellent biocompatibility, liposomes are used in several biomedical applications including drug delivery, gene therapy, and vaccine delivery where these phospholipid vesicles can encapsulate hydrophobic or hydrophilic biomolecular cargo.^8−12^ Computational and experimental investigations of translocations of drug-like molecules through biological membranes are crucial for designing different drug-delivery systems.^13^ Moreover, studying the adsorption and transport properties of drug-like molecules through a phospholipid bilayer in the presence of other drug molecules gives key information on potential drug–drug interactions, where one drug can interact with another drug to change its safety or effectiveness in living organisms.^14−16^

Several examples highlight the growing role of liposome systems for drug-delivery applications as a subset of nanomedicine.^17,18^ Recently, lectin-conjugated liposomes were used as biocompatible and bioadhesive drug carriers to encapsulate various classes of drug molecules for rapid binding to oral epithelial cells on the timescale of minutes, as well as for sustained drug release on the timescale of days.^19^ In another study, different types of azithromycin-loaded liposomes were investigated for treatment of skin infections caused by methicillin-resistant *Staphylococcus aureus* strains.^20^ The incorporation of two drugs, daunorubicin and 6-mercaptopurine, into liposomes demonstrated improved chemotherapeutic applications in the treatment of leukemia.^21^ The interactions between these drugs in solution and inside the liposomes were monitored spectroscopically after phospholipase-mediated liposome lysis, showing a synergistic effect in cell culture studies resulting in increased effectiveness and decreased cytotoxicity.^21^ Additionally, lipid nanoparticle formulations are currently being used for encapsulation and targeted delivery of spike-protein mRNA in COVID-19 vaccines, such as the Pfizer and Moderna vaccines, that are being widely administered for combating the ongoing global pandemic.^22−24^ Both the Pfizer and Moderna COVID-19 vaccines utilize lipid nanoparticles composed of 1,2-distearoyl-sn-glycero-3-phosphocholine phospholipids, which is closely related to 1,2-dioleoyl-*sn*-glycero-3-phosphocholine (DOPC) both chemically and structurally. Understanding potential drug–drug interactions at lipid bilayer interfaces is crucial in the further development of liposome-based and lipid nanoparticle-based drug-delivery applications, especially for ensuring the overall safety and effectiveness of these powerful emerging nanomedicine technologies.

In our previous work, time-resolved second harmonic generation (SHG) spectroscopy was used to investigate molecular adsorption and transport kinetics of drug-like organic dye molecules in different types of liposomes prepared in aqueous solution.^25−27^ In our first study, comparisons of adsorption and transport of malachite green (MG) and methyl green (MetG) dye molecules in 1,2-dioleoyl-sn-glycero-3-phospho-(1′-rac-glycerol) (DOPG), dioleoyl-sn-glycero-3-phospho-l-serine (DOPS), trimethyl quinone-1,2-dioleoyl-sn-glycero-3-phosphoethanolamine (QPADOPE), and DOPC liposomes under different buffer and salt conditions highlighted the influence of several interrelated factors on molecular translocation such as electrostatic interactions, the molecular structures of the lipid headgroups, effects from buffer concentrations, adsorbate–adsorbate repulsions, and ion-pair formation.^25^ In our second study, time-dependent SHG measurements were combined with molecular dynamics (MD) simulations to elucidate fundamental events associated with adsorption and transport of the small molecular cation, malachite green isothiocyanate (MGITC), in comparison to MG in DOPG, DOPC, DOPS, and QPADOPE colloidal liposomes, focusing on changes due to added chemical functional group isothiocyanate.^26^ In our group’s most recent work, SHG spectroscopy and MD simulations were used to study molecular adsorption and transport of MG at the surface of DOPG liposomes in water at different temperatures to determine the thermodynamic properties of adsorption enthalpy and adsorption entropy at the bilayer surface.^27^ In the current study, presented here, we build on our previous work to investigate changes to molecular translocation caused by the presence of a drug molecule acetaminophen (APAP).

APAP, also known as *N*-acetyl-p-aminophenol and paracetamol, is one of the most commonly used over-the-counter drugs for pain and fever relief. APAP is very safe and effective when used as directed.^28−30^ However, despite its wide and common analgesic and antipyretic uses, APAP also shows a variety of side effects and toxicities with potential for overdose when used incorrectly. APAP overdose is the most common cause of acute liver failure and the leading cause of chronic liver damage requiring liver transplantation in developed countries.^29−31^ In animal studies, APAP overdose induced a dramatic change of many phosphatidylcholine and phosphatidylethanolamine species in the plasma membrane of liver cells, resulting in damaged hepatocytes and interference with phospholipid metabolism.^32^ Studies have shown that APAP can interact with zwitterionic phospholipid bilayers, leading to altered membrane fluidity, rigidity, permeability, and morphology.^33−35^ Understanding the unique effects of APAP on mammalian cells, particularly related to molecular-level details of the drug’s influences on physicochemical properties of the cell-membrane integrity and fluidity, is important for mitigating potential toxicity and for developing safer therapeutic treatments.^33^ Additionally, studying changes in molecular adsorption and transport properties of small, drug-like molecular probes with liposome bilayers both with and without added APAP can form the basis for fundamental research on potential drug–drug interactions at cell membranes and in liposome-based drug-delivery applications.

SHG is a powerful, nondestructive, nonlinear spectroscopic technique which can be applied to characterize surfaces and interfaces of colloidal nanoparticles, microparticles, and liposomes.^36,37^ SHG is a frequency-doubling process where two photons of frequency *ω* are added coherently to generate a third photon of frequency 2ω.^25,38^ In the dipole approximation, SHG is forbidden in bulk media with centrosymmetric symmetry, such as in isotropic solutions, while SHG is allowed at surfaces where the symmetry is broken.^36,39,40^ SHG measurements have been used to investigate freely adsorbing molecules at the surface of colloidal nanoparticles as well as the adsorption and transport kinetics at phospholipid bilayer membranes in liposomes and living cells,^25,26,41−45^ where most of this research focuses on cationic dyes such MG,^25,27,43^ MGITC,^26^ and hemicyanine^46^ because of their strong SHG signals when adsorbed to the outer membrane surface.^42,47^ The triphenylmethane dyes of MG, MGITC, and brilliant green also have very low two-photon fluorescence signals because of their ultrafast excited-state relaxation dynamics,^48−50^ making them excellent SHG-active probes for liposome studies. After adsorption, these molecules can transport through the bilayer membrane and adsorb onto the inner surface of the liposome with an opposite orientation compared to dye molecules on the outer surface.^42,46,51^ Because the lipid bilayer thickness is approximately 5 nm, which is much smaller than the SHG coherence length, the second harmonic polarizations of the oppositely oriented molecules on the inner and outer surfaces of the membrane effectively cancel resulting in a decrease in SHG intensity.^36,47^ Therefore, the time-dependent increase and subsequent decrease in the SHG signal provide surface-specific information on the molecular adsorption and transport properties of these SHG-active probe molecules at the liposome bilayer interface.^27,41,52^ The SHG electric field *E*_SHG_ generated at frequency 2ω is linearly proportional to the difference in the population of dye molecules on the outer surface *N*_0_ and the inner surface *N_i_* and given by the equation,^47,53^
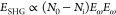
1where *E*_ω_ is the incident optical electric field at frequency ω. The measured intensity of the SHG signal *I*_SHG_ at frequency 2ω is given by *I*_SHG_ = *E*_SHG_^2^.

In this study, SHG spectroscopy is used to monitor the adsorption and transport kinetics of the drug-like dye molecule MGITC in DOPC liposomes in water both with and without added APAP. DOPC is a common phospholipid in mammalian cell membranes and has been used in several drug-delivery applications.^54−56^ The adsorption of MGITC to DOPC liposomes is driven by electrostatic and dipole–dipole interactions between the positively charged MGITC molecules and the zwitterionic phospholipid membranes.^26^ Time-resolved SHG signals are used to determine the adsorption equilibrium constants, adsorbate site densities, and molecular transport times, characterizing the detailed molecular interactions with the liposome surface. Additionally, these measurements are repeated after adding APAP to determine corresponding changes to these molecular interactions caused by the presence of this added drug molecule. These surface-sensitive nonlinear optical measurements of molecular adsorption and transport at colloidal liposome interfaces provide a detailed foundation for understanding molecular interactions with phospholipid bilayers and drug–drug interactions for designing safe and effective drug-delivery applications.

## Experimental Section

### Synthesis and Characterization of Liposomes

The synthesis of large unilamellar vesicles of DOPC liposomes has been previously reported.^33^ DOPC lyophilized powder, purchased from NOF America Corporation, and APAP powder, purchased from Spectrum Chemical MFG Corp, are dissolved in HPLC-grade chloroform (0.4 mg/mL), purchased from Sigma-Aldrich. After dissolving, the DOPC and APAP are mixed to obtain specific molar ratios of 1:0, using 75 μM DOPC and 0 μM APAP, and 3:1, using 75 μM DOPC and 25 μM APAP. The samples are placed under a nitrogen stream until most of the solvent is evaporated and then placed in a vacuum oven overnight to remove the remaining organic solvent traces. The obtained dry lipid cakes are hydrated with 5 mL of ultrapure water for each sample. The vesicle suspensions then undergo eight freeze–thaw cycles at −20 and 50 °C in 10 min intervals. Finally, the vesicles are extruded using an Avanti Mini-Extruder with 100 nm polycarbonate membranes passing the vesicle suspension 33 times through the membrane to obtain large unilamellar vesicles with diameters of approximately 100 nm. After extrusion, 95 mL of ultrapure water is added to each sample, making a total of 100 mL per sample. The DOPC liposomes are in the fluid phase at ambient temperature, with a transition melting temperature *T*_m_ of −16.5 °C where the ordered gel phase changes to the more disordered fluid phase.^57^ Additional characterization of DOPC liposomes is discussed in the Supporting Information. The molecular structures of DOPC, MGITC, and APAP are also shown in Figure S1.

### SHG Setup

The SHG spectroscopy setup has been described previously.^25,26,58^ Briefly, a titanium:sapphire oscillator laser output centered at 800 nm with 75 fs pulses at a repetition rate of 80 MHz is attenuated to 1 W using a neutral density filter and is focused into a 1 cm quartz cuvette containing the colloidal DOPC liposomes in aqueous solution. The SHG signal is collected in the forward direction and is detected as a function of time using a high-sensitivity spectroscopy charge-coupled device connected to a monochromator spectrograph. Additional details of the SHG setup are provided in the Supporting Information.

## Results and Discussion

**Figure 1:**
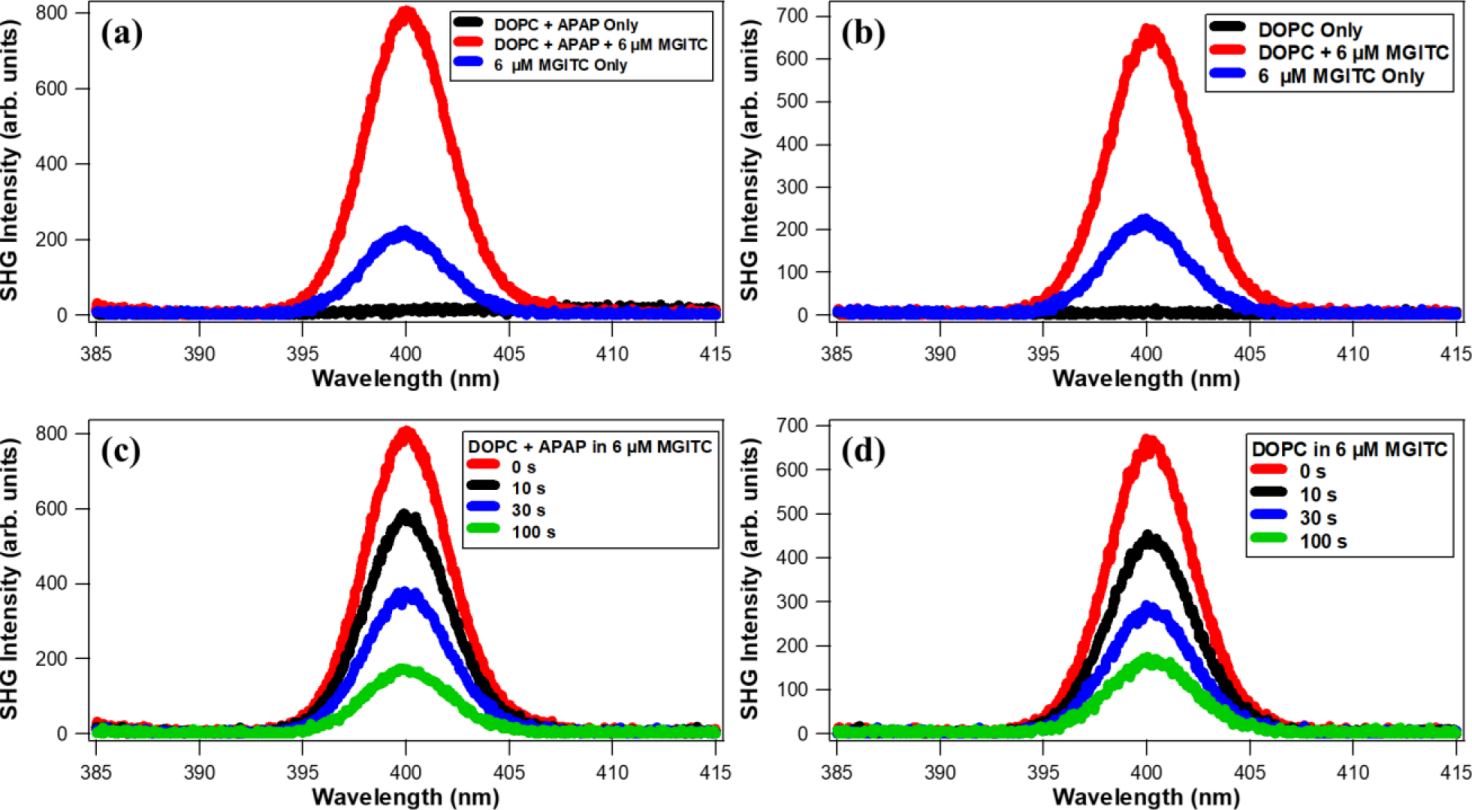
(a) SHG spectra of DOPC liposomes with APAP with and without 6 μM MGITC compared to 6 μM MGITC alone. (b) SHG spectra of pure DOPC liposomes with and without 6 μM MGITC compared to 6 μM MGITC alone. (c) SHG spectra of DOPC liposomes with APAP at various times after the addition of 6 μM MGITC. (d) SHG spectra of pure DOPC liposomes at various times after the addition of 6 μM MGITC.

Figure 1 shows representative SHG spectra of the DOPC liposomes at 75 μM lipid concentration with and without 25 μM APAP and 6 μM MGITC in water. MGITC is a hydrophobic, drug-like cationic molecule which has an absorption near 400 nm that provides a resonant enhancement of the SHG signal when adsorbed to the liposome surface.^26^ The extinction spectrum of MGITC in water is displayed in the Supporting Information. As shown in Figure 1a,b, the SHG intensity of the DOPC liposomes with and without APAP is negligible before MGITC is added. Upon addition of 6 μM MGITC solution to the liposomes, a large SHG peak centered at 400 nm is observed with a full-width at half-maximum of approximately 4.7 nm. These results are compared to the corresponding spectrum from 6 μM MGITC in water alone, without liposomes present, where the 400 nm signal originates from hyper-Rayleigh scattering (HRS).^25,26,59,60^ The SHG signal of MGITC in DOPC liposomes is approximately 3.5 times greater than the HRS from MGITC alone because of adsorption of MGITC at the liposome surface, in general agreement with our previous observations.^26^

As shown in Figure 1c,d, the SHG signal from 6 μM MGITC added to the DOPC liposomes both with and without APAP decreases as a function of time, which is caused by the transport of MGITC molecules across the phospholipid bilayer.^26,53^ According to our previous work, MG, which has a similar molecular structure to MGITC, demonstrates no adsorption or transport in DOPC liposomes.^25^ In this case, MGITC adsorbs and transports through the DOPC liposome membrane much more efficiently than MG because of the isothiocyanate group in MGITC molecules and the added dipole–dipole interactions with the zwitterionic DOPC bilayer.^26^

**Figure 2:**
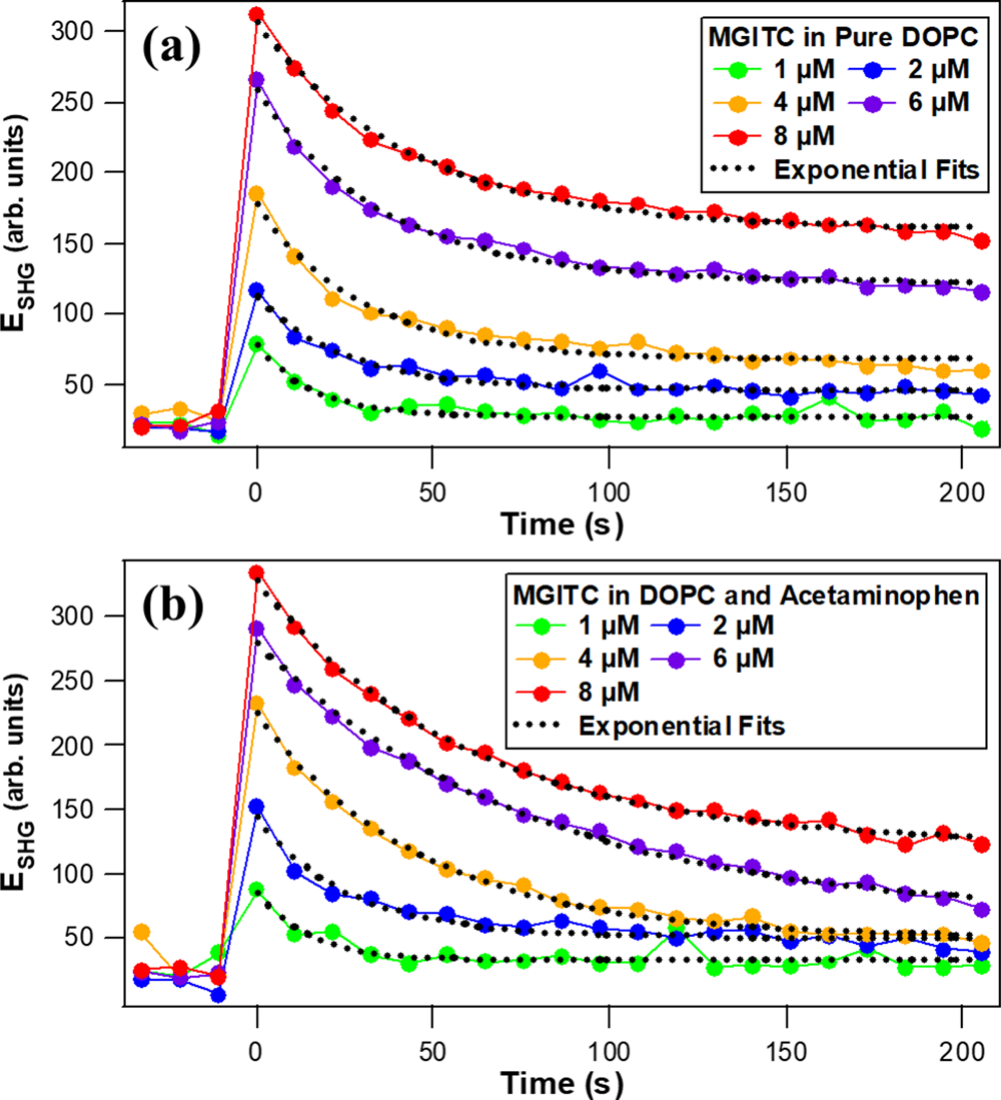
Representative SHG time profiles upon addition of various concentrations of MGITC to (a) pure DOPC liposomes and (b) DOPC liposomes with added APAP in water along with best fits (dotted black lines).

The SHG time traces of MGITC added to DOPC liposomes with and without APAP in water at various dye concentrations are shown in Figure 2. A very rapid rise in the SHG signal intensity occurs at time zero, when MGITC is added to the liposome sample followed by a gradual decrease in intensity as the MGITC molecular transport process takes place until reaching equilibrium. Very quickly after the dye solution is added to liposomes, on a timescale faster than our current experimental resolution, the adsorption of MGITC molecules onto the outer surface of DOPC liposomes occurs along with alignment to an orientational distribution at the interface, causing an abrupt rise in SHG intensity from an enhanced χ^(2)^ second-order nonlinear susceptibility at the liposome surface. The decrease of the SHG signal is the result of MGITC molecules migrating across the DOPC membrane and adsorbing at the inner bilayer surface with an opposite orientation compared to the outer surface, causing a cancelation and an overall decrease of the SHG signal.^53,61,62^

**Figure 3:**
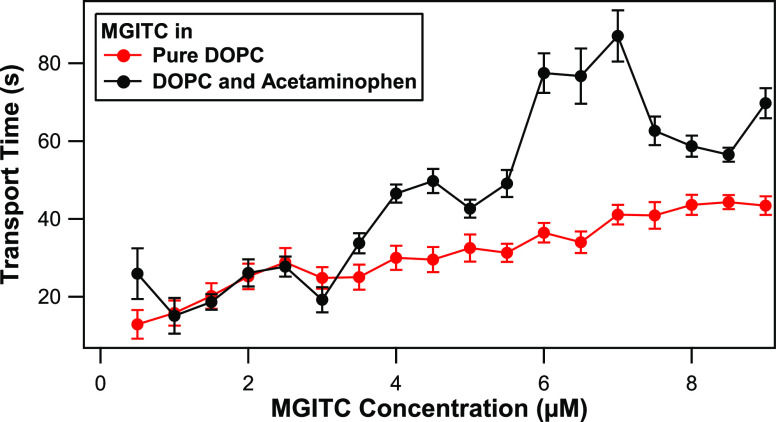
Transport times as a function of MGITC concentration for pure DOPC liposomes (red circles) and DOPC liposomes with APAP (black circles) in water.

The experimental SHG time traces are plotted in Figure 2 for DOPC liposomes with and without APAP, and the results are fit to single exponential functions given by,
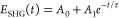
2to measure the molecular transport times τ, where *E*_SHG_(*t*) is the SHG electric field at experimental time *t* after MGITC addition, and *A*_0_ and *A*_1_ are proportionality constants. Each SHG time trace uses a fresh liposome sample. The obtained transport times are plotted as a function of the MGITC concentration for each liposome sample, as shown in Figure 3. The transport time of MGITC is the same, to within experimental uncertainty, for pure DOPC liposomes and DOPC liposomes with APAP for MGITC concentrations of 1 to 2.5 μM. However, for MGITC concentrations greater than 3 μM, the transport lifetime is longer in DOPC liposomes with APAP than in pure DOPC liposomes. The transport times obtained for each liposome sample at different MGITC concentrations are tabulated in Table S1 in the Supporting Information.

**Figure 4:**
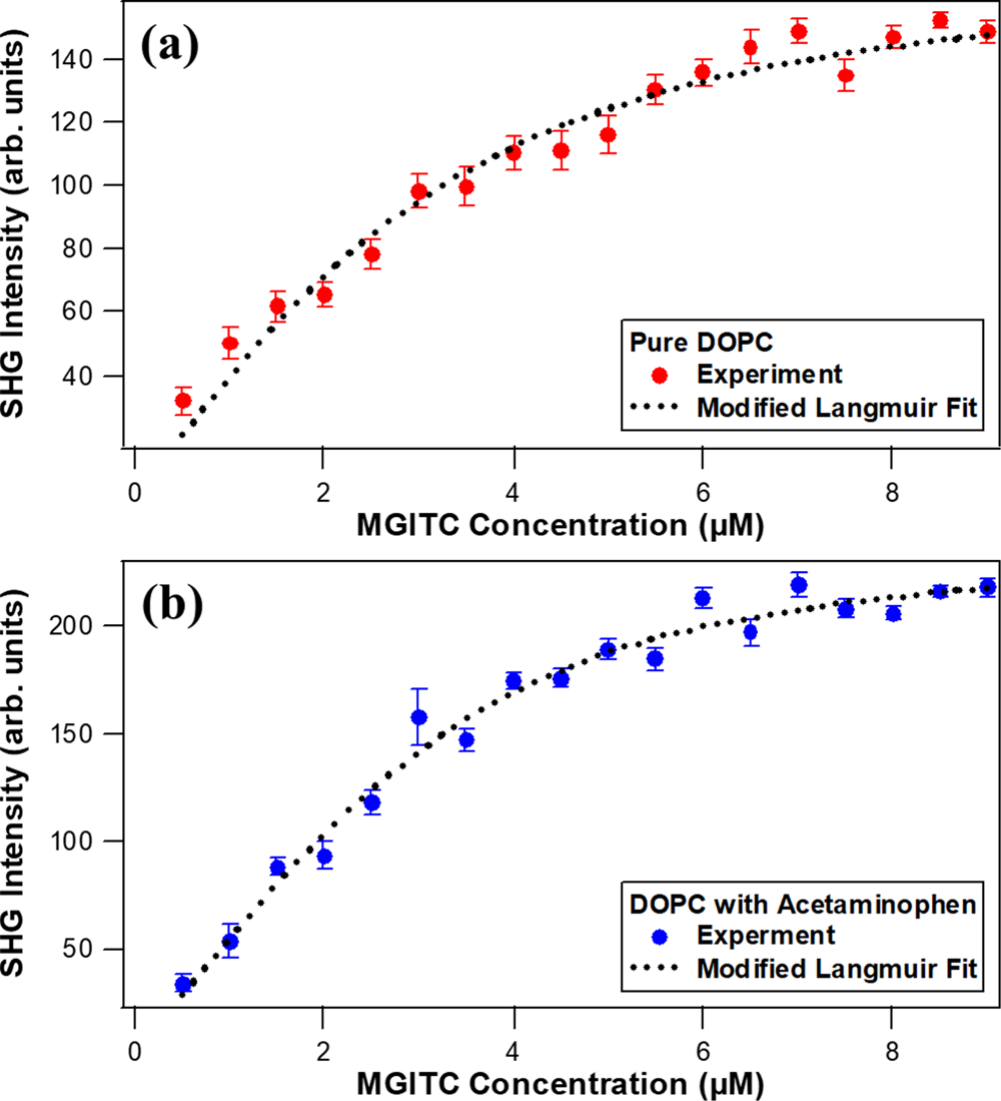
Experimental adsorption isotherms for MGITC with (a) pure DOPC liposomes and (b) DOPC liposomes with APAP. Dotted lines are best fits from the modified Langmuir model.

The sudden rise in the SHG signal upon MGITC addition to the DOPC liposome samples at time zero, as shown in Figure 2, occurs because of the adsorption of MGITC molecular ions to the outer surface of the bilayer. By plotting the SHG signal at time zero for each MGITC concentration, the adsorption isotherms are obtained, as shown in Figure 4a,b for pure DOPC liposomes and for DOPC liposomes with APAP, respectively. The SHG intensities are fit using the modified Langmuir model^26,63,64^ to obtain the corresponding adsorption equilibrium constants and the adsorbate site densities. The modified Langmuir model is an extended form of the Langmuir model, which accounts for bulk depletion of the adsorbate because of a large cumulative liposome surface area. This model assumes that freely adsorbing molecules form a single monolayer with a corresponding maximum adsorbate site density at the liposome surface.^65^ The modified Langmuir model is given by
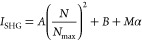
3
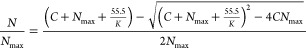
4where *N i*s the concentration of MGITC dye molecules adsorbed on the DOPC liposome surface, *N*_max_ is the maximum adsorption site concentration, *A* is the SHG intensity at saturation, *B* is the baseline offset due to the SHG signal from liposomes in water without the addition of the dye, *M* is the concentration of free dye molecules in solution, α is the slope obtained from the plot of SHG intensity of dye alone as a function of concentration *C*, 55.5 is the molar concentration of water, and *K* is the adsorption equilibrium constant. The adsorption isotherms are fit using three fit parameters, *A*, *N*_max_, and *K*. The experimental data are corrected to account for the contribution from HRS from free MGITC molecules in water, which is displayed in the Supporting Information, to give the α values. The free energy of adsorption is obtained using *ΔG* = – *RT* ln *K*. The modified Langmuir fits are shown as dotted black lines in Figure 4a,b for MGITC added to the DOPC liposomes with and without APAP, respectively. The corresponding fit parameters and the corresponding free energies are summarized in Table 1 for pure DOPC liposomes and DOPC liposomes with APAP. By dividing the lipid concentrations by the *N*_max_ values, the lipids per adsorption site are obtained for each sample, and these results are also listed in Table 1.

**Table 1:** List of Variables and Fitting Parameters Obtained from the Modified Langmuir Model with Liposomes of Pure DOPC and DOPC with APAP

sample	*A*	*K*	*N*_max_ (μM)	lipid/site	Δ*G* (kcal mol^–1^)
pure 75 μM DOPC	174 ± 2	(5 ± 1) × 10^7^	2.9 ± 0.5	25.9 ± 4.5	–10.3 ± 0.1
75 μM DOPC with 25 μM APAP	241 ± 3	(9 ± 1) × 10^7^	3.6 ± 0.3	20.8 ± 1.7	–10.6 ± 0.1

As shown in Table 1, the equilibrium constants of MGITC adsorbing to the liposome samples are (5 ± 1) × 10^7^ and (9 ± 1) × 10^7^ for pure DOPC and for DOPC with APAP, respectively. This shows that APAP increases the electrostatic and dipole–dipole attractions of MGITC adsorption to the liposome surface. The corresponding free energy of adsorption is −10.3 ± 0.1 and −10.6 ± 0.1 kcal/mole for DOPC liposomes with and without added APAP, respectively. The maximum adsorption site concentration *N*_max_ of MGITC adsorbing to pure DOPC liposomes is 2.9 ± 0.5 μM compared to the corresponding *N*_max_ value of 3.6 ± 0.3 μM for DOPC liposomes with added APAP. These values are consistent with the interpretation of increased adsorption of MGITC when APAP is present. The corresponding number of lipid molecules per adsorption site (lipid/site) is 25.9 ± 4.5 and 20.8 ± 1.7 for pure DOPC liposomes and DOPC liposomes with added APAP, respectively. A positive attraction of MGITC to APAP can also have synergistic effects, where increased MGITC concentrations at the liposome surface can draw more APAP from the aqueous bulk to the lipid bilayer. The SHG intensity *A* at adsorbate saturation is 174 ± 2 and 241 ± 3 for liposomes of pure DOPC and DOPC with APAP, respectively. The ratio of  provides a direct comparison of the SHG signal per adsorbate surface coverage, with values of 21 ± 7 and 19 ± 3 μM^–2^ for liposomes of pure DOPC and DOPC with APAP, respectively. Although the liposomes with APAP have greater SHG signals at saturation, the SHG signal is shown to scale quadratically according to the *N*_max_ adsorbate surface coverage, to within experimental uncertainty, indicating a similar MGITC angular distribution at the surface for both liposome samples. Overall, these SHG measurements show that APAP increases MGITC adsorption to the DOPC liposome surface resulting in larger magnitudes of adsorption free energy and higher adsorption site densities.

A comparison of the adsorption isotherm measurements with the molecular transport times highlights the complicated interactions between MGITC, DOPC, and APAP. APAP is a neutral, weak acid with a p*K*a value of 9.5 and a relatively low lipophilicity.^66^ The octanol/water partition coefficient for APAP has a log*P* value of 0.38,^66^ indicating that APAP is more soluble in water than in octanol. MGITC has a higher log*P* value of 1.67, showing a higher lipophilicity.^67^ Previous SHG studies of liposomes observed that the transport times of the similar dye molecule MG increase because of effects such as ion pairing with anions such as citrate and chloride^43^ and higher membrane rigidity by the addition of cholesterol^68^ or by decreased temperatures.^27^ However, in our results presented here, the transport times of MGITC at concentrations of 1 to 2.5 μM are approximately the same for the liposome samples both with and without added APAP, to within experimental uncertainty, while the transport times are significantly longer when APAP is added at higher MGITC concentrations of 3.5 μM and above, closer to the adsorption saturation level. This indicates a thresholding effect, where larger interactions between APAP and MGITC are activated at higher MGITC concentrations. Our recent investigation of DOPC liposomes with APAP using small-angle X-ray and neutron scattering showed a slight decrease of the diameter and that the number of lamellae did not change after the addition of APAP, while cryo-transmission electron microscopy revealed increased heterogeneity after the addition of APAP, mirrored by the occurrence of irregular shape morphologies.^33^ Neutron spin echo spectroscopy revealed a strong decrease of the bending modulus and the space explored by the lipid tails with increasing APAP concentration.^33^ An increase in DOPC membrane fluidity is expected to decrease the MGITC transport time. However, the APAP in the lipid bilayer can also hinder transport because of attractive electrostatic and dipole–dipole interactions between APAP and MGITC, or through more complicated interactions involving APAP with the DOPC membrane, creating counter-balancing effects to the increased membrane fluidity. These results highlight the complicated chemical and physical interactions that can occur both at the surface and within phospholipid bilayers, which depend on many factors including electrostatics, hydrophobic–hydrophilic interactions, dipole–dipole interactions, and membrane fluidity. In addition, this research demonstrates a new experimental framework for investigating chemical interactions with drug molecules at phospholipid bilayers using SHG spectroscopy to study molecular translocation processes for advancing potential drug-delivery applications.

## Conclusions

The effect of APAP on the adsorption and transport properties of MGITC in DOPC liposomes is investigated using time-dependent, surface-sensitive SHG spectroscopy. The SHG results are used to determine the adsorption equilibrium constants, adsorption site densities, and transport kinetics of MGITC at the DOPC liposome surface, both with and without APAP. The molecular transport of MGITC is found to be more rapid in pure DOPC liposome samples compared to DOPC liposomes with added APAP, but this effect occurs predominantly at higher MGITC concentrations. The SHG adsorption isotherms are fit using the modified Langmuir model, showing that the free energy of adsorption increases slightly in magnitude along with a corresponding increase in adsorption site density when APAP is added to the DOPC liposomes. These results highlight the complicated molecular interactions between MGITC, APAP, and the DOPC liposome surface, with factors that depend on electrostatics, dipole–dipole interactions, hydrophobic/hydrophilic interactions, and membrane fluidity. This research also demonstrates a pathway for investigating fundamental properties of molecular translocation at biological membranes, including drug–drug interactions, that are important in the development of drug-delivery technologies.

## Supporting Information Available

Additional characterization of DOPC liposomes, the SHG setup, and tabulated fitting parameters (PDF)
