# Supplementary material for: Influence of Acetaminophen on Molecular Adsorption and Transport Properties at Colloidal Liposome Surfaces Studied by Second Harmonic Generation Spectroscopy
Source: Langmuir. 2022 Mar 17;38(12):3852–9. doi: 10.1021/acs.langmuir.2c00086 (PMC8969770; doi:10.1021/acs.langmuir.2c00086)
Supplement: Supplementary file 1 — la2c00086_si_001.pdf [file la2c00086_si_001.pdf]

Supporting Information for

Influence of Acetaminophen on Molecular Adsorption and Transport Properties at Colloidal Liposome Surfaces Studied by Second Harmonic Generation Spectroscopy

Asela S. Dikkumbura,[†] Alexandra V. Aucoin,[†] Rasidah O. Ali,[†] Aliyah Dalier,[§] Dylan W. Gilbert,[§] Gerald J. Schneider,[†,‡] and Louis H. Haber,[†,*]

[†] Department of Chemistry and [‡] Department of Physics and Astronomy, Louisiana State University, Baton Rouge, Louisiana 70803, USA.

[§] Southeastern Louisiana University, Hammond, Louisiana 70402, USA.

Additional Characterizations of Liposomes

The molecular structures of 1,2-dioleoyl-*sn*-glycero-3-phosphocholine (DOPC), malachite green isothiocyanate (MGITC), and acetaminophen (APAP) are shown in Figure S1.

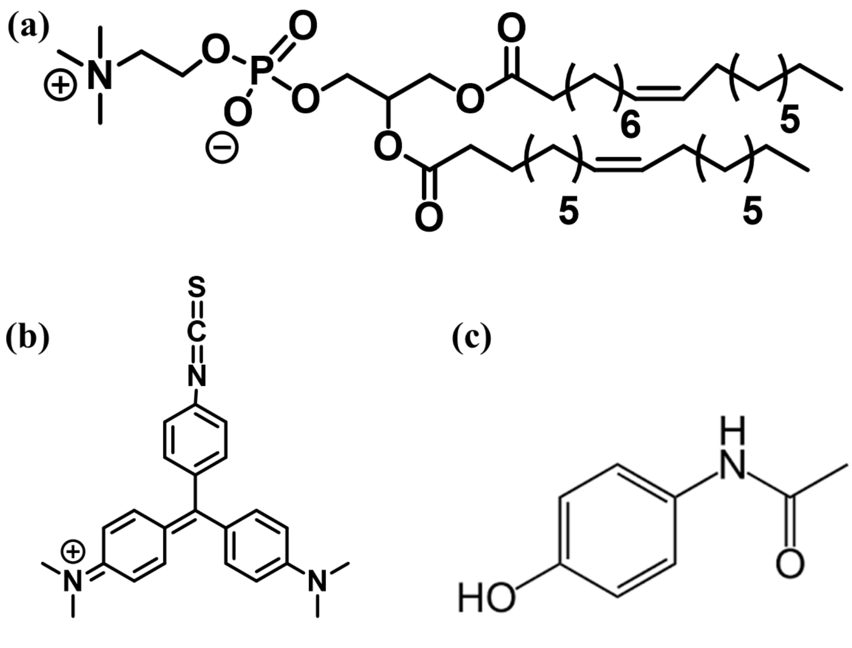

Figure S1. Molecular structures of (a) DOPC, (b) MGITC, and (c) APAP.

Figure S2 displays the dynamic light scattering (DLS) size distributions and zeta potential measurements for 75 μM DOPC liposomes with and without 25 μM APAP. The sizes are determined to be 109.7 ± 0.9 nm and 104.6 ± 1.3 nm, and the zeta potentials are −28.0 ± 7.3 mV and −25.8 ± 5.8 mV for pure DOPC liposomes and for DOPC liposomes with APAP, respectively. The polydispersity index values are 0.10 ± 0.02 and 0.11 ± 0.02 for liposomes of pure DOPC and DOPC with APAP, respectively. Zeta potential and DLS measurements are obtained using a Zetasizer Nano ZS from Malvern Instruments Inc.

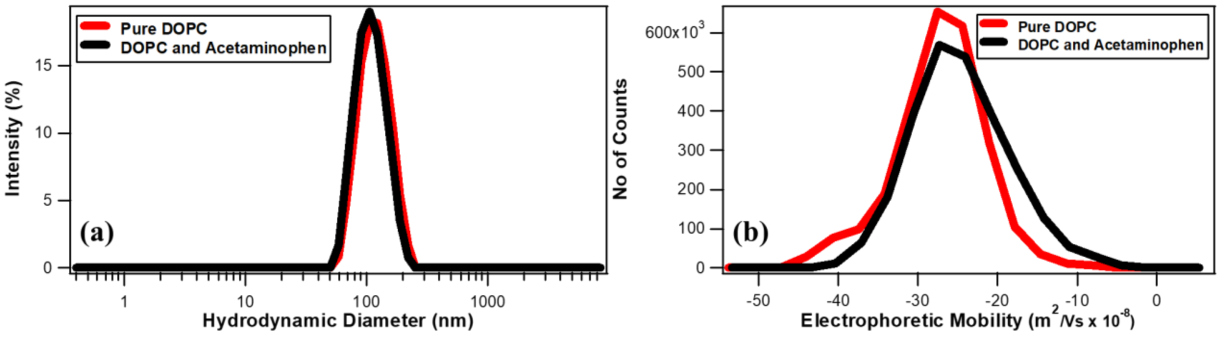

Figure S2. (a) DLS size distributions and (b) zeta potential measurements for DOPC liposomes with and without APAP.

Additional Details on Second Harmonic Generation (SHG) Setup

The home-built second harmonic generation (SHG) setup is shown in Figure S3. An optical filter is placed in front of the cuvette to remove any SHG light from the optical setup prior to the colloidal liposome sample. Another filter is placed after the sample to remove the fundamental 800 nm light, while allowing the SHG signal through. The SHG signal is collected in the forward direction and refocused to the monochromator spectrograph connected to a high-sensitivity charge-coupled device (CCD) detector. This optical setup allows for the detection of the SHG signal as a function of time to capture the dynamics of MGITC adsorption and transport through the phospholipid liposome bilayer. A data acquisition program collects 5 SHG spectra and 5

background spectra using a computer-controlled beam block in repeating iterations with acquisition times of 1.0 s.[1-2] All SHG measurements are done at room temperature (22 °C).

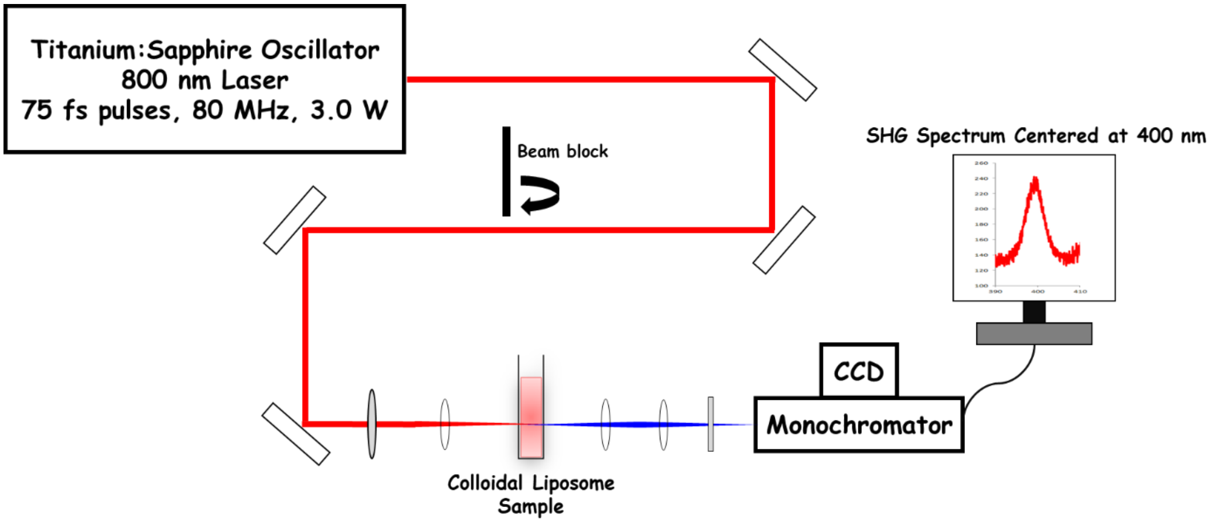

Figure S3. Home-built SHG spectroscopy setup.

Extinction Spectrum and Additional SHG Measurements of MGITC

The extinction spectrum of MGITC in water is shown in Figure S4. Extinction measurements are done using a home-built UV-Vis spectrometer, as described previously.[3-4]

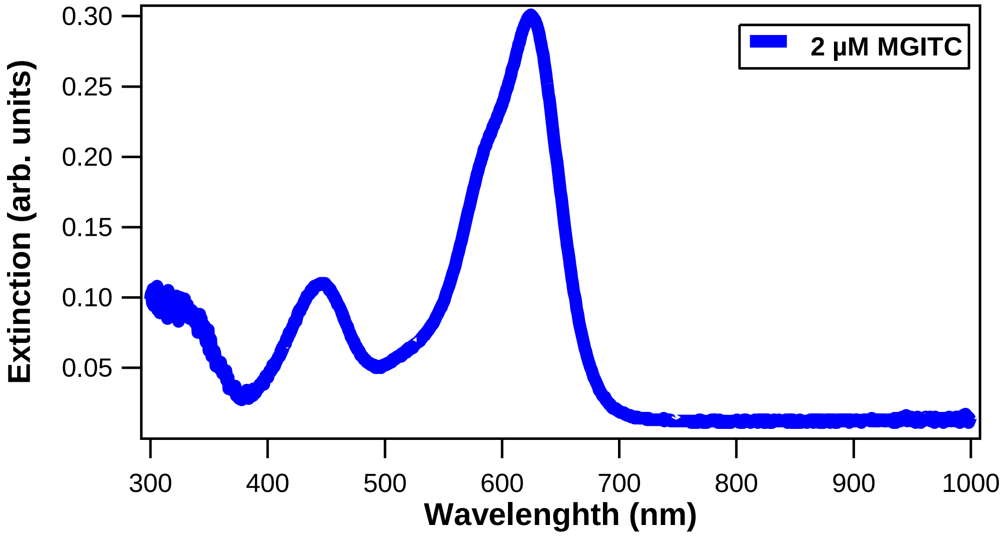

Figure S4. The extinction spectrum of 2 μM MGITC dye in water.

The experimental data are corrected to account for the contribution from hyper-Rayleigh scattering (HRS) from the free MGITC molecules in water. The HRS signal as a function of MGITC dye concentration is shown in Figure S5 along with a best linear fit.

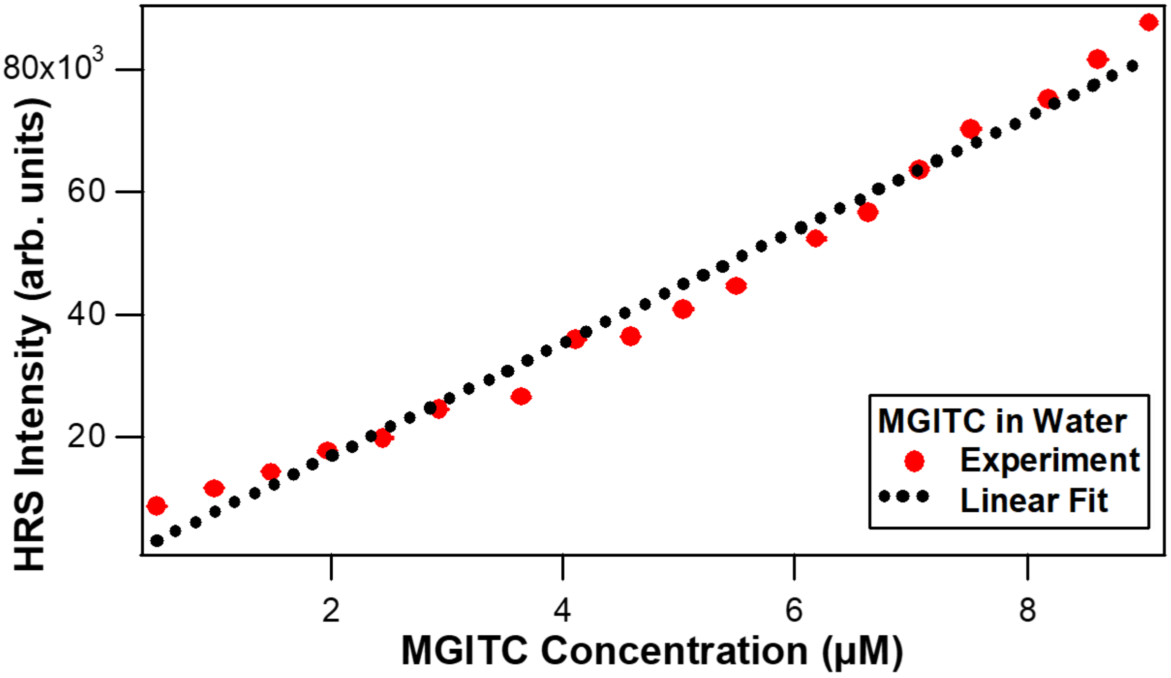

Figure S5. HRS signal intensity of water as a function of added MGITC concentration (red circles) compared to the linear fit (dotted black line).

Additional Details on SHG Time Profile Fits

The transport times τ and proportionality constants A_0 and A_1 obtained from the SHG time trace fits using Equation (2) in the paper for the DOPC liposome samples with and without APAP are listed in Table S1.

Table S1. Transport times and proportionality constants of MGITC in DOPC samples with and without acetaminophen.

[MGITC] (μM)	Condition	τ (s)	A_0	A_1
0.5	DOPC	12.9 ± 3.7	28.3 ± 1.1	31.9 ± 4.2
0.5	DOPC and APAP	25.9 ± 6.5	22.7 ± 1.4	33.8 ± 4.1
1.0	DOPC	15.8 ± 3.7	27.9 ± 1.4	50.4 ± 5.0
1.0	DOPC and APAP	15.1 ± 4.6	33.0 ± 2.1	53.4 ± 7.8
1.5	DOPC	20.2 ± 3.3	43.1 ± 1.5	62.1 ± 5.0
1.5	DOPC and APAP	18.6 ± 2.0	32.3 ± 1.3	88.2 ± 4.5
2.0	DOPC	25.2 ± 3.3	47.0 ± 1.4	65.7 ± 4.2
2.0	DOPC and APAP	26.1 ± 3.5	51.0 ± 2.1	93.5 ± 6.2
2.5	DOPC	28.8 ± 3.7	56.4 ± 1.8	78.4 ± 4.8
2.5	DOPC and APAP	27.7 ± 2.6	53.8 ± 1.9	118.0 ± 5.4
3.0	DOPC	24.8 ± 2.8	49.4 ± 1.8	98.4 ± 5.5
3.0	DOPC and APAP	19.2 ± 3.2	39.5 ± 3.8	157.7 ± 12.9
3.5	DOPC	25.0 ± 3.2	75.0 ± 2.1	100.0 ± 6.3
3.5	DOPC and APAP	33.7 ± 2.6	65.2 ± 2.1	147.0 ± 5.2
4.0	DOPC	30.3 ± 3.1	68.0 ± 2.1	110.7 ± 5.5
4.0	DOPC and APAP	46.5 ± 2.3	50.9 ± 2.1	174.7 ± 3.6
4.5	DOPC	29.5 ± 3.2	88.9 ± 2.2	111.4 ± 5.8
4.5	DOPC and APAP	49.7 ± 3.1	73.7 ± 2.7	176.1 ± 4.3
5.0	DOPC	32.5 ± 3.5	101.1 ± 1.3	116.2 ± 5.9
5.0	DOPC and APAP	42.6 ± 2.3	65.3 ± 2.3	189.5 ± 4.3
5.5	DOPC	31.3 ± 2.3	101.3 ± 1.8	130.3 ± 4.6
5.5	DOPC and APAP	49.1 ± 3.5	82.8 ± 3.3	185.0 ± 5.3
6.0	DOPC	36.4 ± 2.5	122.9 ± 1.9	136.1 ± 4.2
6.0	DOPC and APAP	77.5 ± 5.1	66.6 ± 5.0	213.3 ± 4.7
6.5	DOPC	34.0 ± 2.8	137.9 ± 2.3	143.9 ± 5.4
6.5	DOPC and APAP	76.7 ± 7.1	99.2 ± 6.3	197.4 ± 6.1
7.0	DOPC	41.1 ± 2.5	113.5 ± 2.0	149.4 ± 3.9
7.0	DOPC and APAP	87.0 ± 6.6	96.4 ± 6.4	219.4 ± 5.7
7.5	DOPC	40.9 ± 3.4	147.4 ± 2.4	135.2 ± 4.8
7.5	DOPC and APAP	62.6 ± 3.7	113.3 ± 3.7	208.5 ± 4.4
8.0	DOPC	43.6 ± 2.6	160.3 ± 2.0	147.3 ± 3.8
8.0	DOPC and APAP	58.7 ± 2.7	122.6 ± 2.7	206.4 ± 3.4
8.5	DOPC	44.3 ± 1.8	162.3 ± 1.4	152.4 ± 2.6
8.5	DOPC and APAP	56.5 ± 1.8	132.6 ± 1.9	216.8 ± 2.6
9.0	DOPC	43.4 ± 2.4	162.4 ± 1.9	148.8 ± 3.5
9.0	DOPC and APAP	69.7 ± 3.9	126.3 ± 9.9	218.2 ± 4.1

References

1. Hamal, P.; Nguyenhuu, H.; Subasinghege Don, V.; Kumal, R. R.; Kumar, R.; McCarley, R. L.; Haber, L. H., Molecular adsorption and transport at liposome surfaces studied by molecular dynamics simulations and second harmonic generation spectroscopy. *The Journal of Physical Chemistry B* **2019,** *123* (36), 7722-7730.
2. Kumal, R. R.; Nguyenhuu, H.; Winter, J. E.; McCarley, R. L.; Haber, L. H., Impacts of salt, buffer, and lipid nature on molecular adsorption and transport in liposomes as observed by second harmonic generation. *The Journal of Physical Chemistry C* **2017,** *121* (29), 15851-15860.
3. Khoury, R. A.; Ranasinghe, J. C.; Dikkumbura, A. S.; Hamal, P.; Kumal, R. R.; Karam, T. E.; Smith, H. T.; Haber, L. H., Monitoring the Seed-Mediated Growth of Gold Nanoparticles Using in Situ Second Harmonic Generation and Extinction Spectroscopy. *The Journal of Physical Chemistry C* **2018,** *122* (42), 24400-24406.
4. Ranasinghe, J. C.; Dikkumbura, A. S.; Hamal, P.; Chen, M.; Khoury, R. A.; Smith, H. T.; Lopata, K.; Haber, L. H., Monitoring the growth dynamics of colloidal gold-silver core-shell nanoparticles using in situ second harmonic generation and extinction spectroscopy. *The Journal of chemical physics* **2019,** *151* (22), 224701.
